# Supplementary material for: Absolute Configuration of Mycosporine-Like Amino Acids, Their Wound Healing Properties and In Vitro Anti-Aging Effects
Source: Mar Drugs. 2019 Dec 31;18(1):35. doi: 10.3390/md18010035 (PMC7024213; doi:10.3390/md18010035)
Supplement: Supplementary file 1 [file marinedrugs-18-00035-s001.pdf]

marine drugs

Supplementary materials

Absolute Configuration of Mycosporine-Like Amino Acids, their Wound Healing Properties and In Vitro Anti-Aging Effects

Maria Orfanoudaki [1,†], Anja Hartmann [1,†,*], Mostafa Alilou [1], Thomas Gelbrich [2], Patricia Blanchard [3], Séverine Derbré [3], Andreas Schinkovitz [3], Pascal Richomme [3], Andreas Hensel [4] and Markus Ganzera [1]

[1] Institute of Pharmacy, Pharmacognosy, University of Innsbruck, Innrain 80-82, Innsbruck 6020, Austria; Maria.Orfanoudaki@uibk.ac.at (M.O.); mostafa.alilou@student.uibk.ac.at (M.A.); markus.ganzera@uibk.ac.at (M.G.)

[2] Institute of Pharmacy, Pharmaceutical Technology, University of Innsbruck, Innrain 52c, Innsbruck 6020, Austria; thomas.gelbrich@uibk.ac.at (T.G.)

[3] SONAS, EA921, University of Angers, SFR QUASAV, Faculty of Health Sciences, Department of Pharmacy, 16 Bd Daviers, 49045, Angers, France; patricia.planchenault@univ-angers.fr (P.P); severine.derbre@univ-angers.fr (S.D.); andreas.schinkovitz@univ-angers.fr (A.S.); pascal.richomme@univ-angers.fr (P.R.)

[4] Institute of Pharmaceutical Biology and Phytochemistry, University of Münster, Corrensstraße 48, D-48149 Münster, Germany, ahensel@uni-muenster.de (A.H.)

* Correspondence: Anja.Hartmann@uibk.ac.at; Tel.: +43 512 507-58430

† These authors contributed equally to this work.

Contents

Figure S1 LC-MS of Marfey´s analysis of compound **1**...3

Figure S2 LC-MS of Marfey´s analysis of compound **2**...3

Figure S3 LC-MS of Marfey´s analysis of compound **3**...4

Figure S4 LC-MS of Marfey´s analysis of compound **4**...4

Figure S5 LC-MS of Marfey´s analysis of compound **5**...5

Figure S6 Experimental ECD spectra of isolated compounds……………………………………………5

Figure S7 Overlayered conformers and population of Boltzmann averaged conformers of compound **1**..6

Figure S8 Overlayered conformers and population of Boltzmann averaged conformers of compound **3**..7

Figure S9 Overlayered conformers and population of Boltzmann averaged conformers of compounds **7**...8

Figure S10 Overlayered conformers and population of Boltzmann averaged conformers of compounds **9**...9

Figure S11 Overlayered conformers and population of Boltzmann averaged conformers of compounds **10**...9

Figure S12 Overlayered conformers and population of Boltzmann averaged conformers of compounds **11**...10

Figure S13 Collagenase inhibitory activity, concentration response curves of all tested MAAs. 11

Figure S14 Dose-effect curves for pentosidine-like AGEs formation in the presence of various concentrations of tested MAAs and rutin and aminoguanidine used as reference compounds. 12

Table S1. Crystallographic data for the hydrate of the shinorine hydrate **1**H..............13

Table S 2 Atomic coordinates (× 10^4) and equivalent isotropic displacement parameters 13

Table S 3 Bond lengths [Å] and angles [°]..14

Table S 4 Anisotropic displacement parameters (Å^2 × 10^3). The anisotropic displacement factor exponent takes the form: $-2\pi^2$ [h^2 a^{*2} U^{11} + ... + 2 h k a* b* U^{12}]. 20

Table S 5 Hydrogen coordinates (× 10^4) and isotropic displacement parameters (Å^2 × 10^3). 22

Table S 6 Hydrogen bonds [Å and °]..23

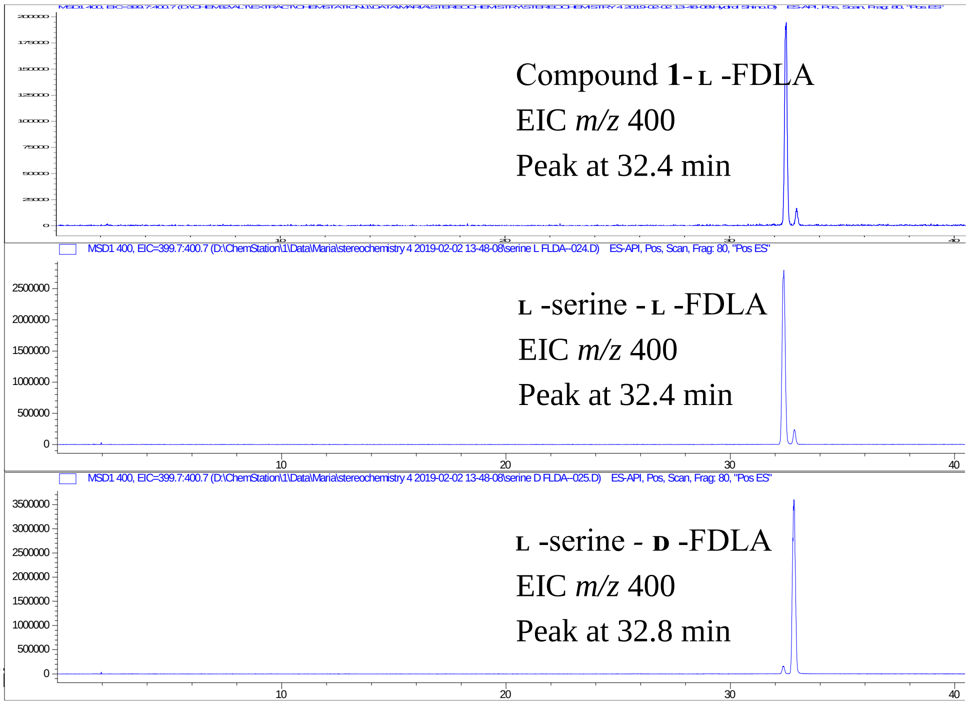

Fi

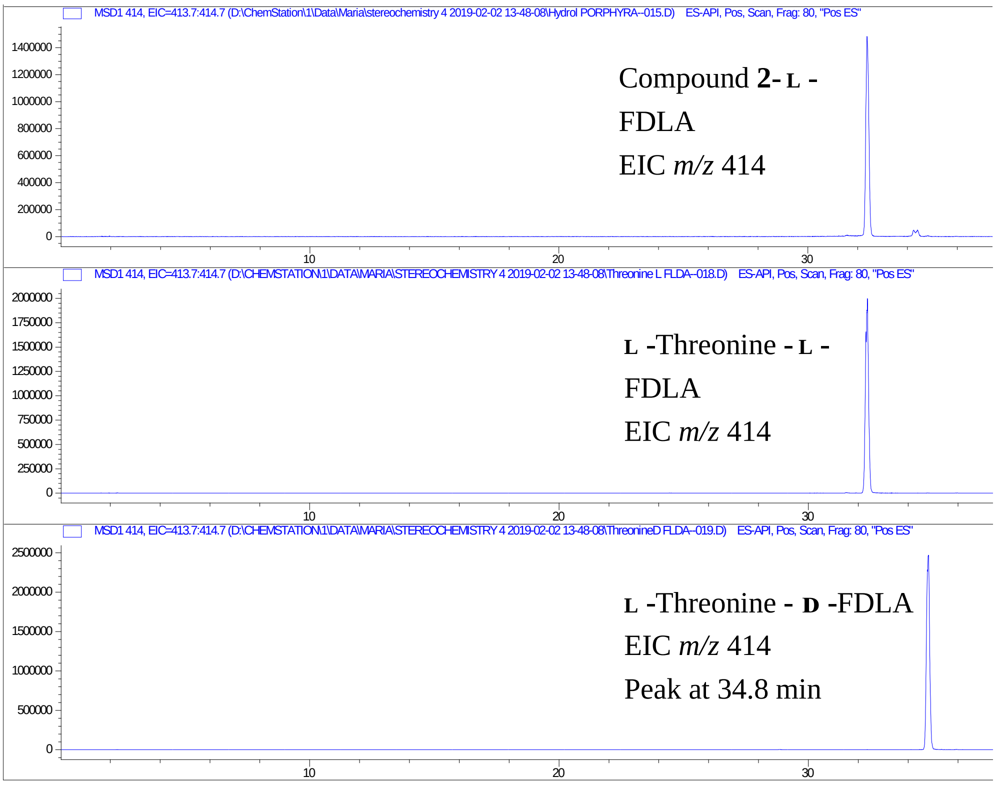

Figure S2 LC-MS of Marfey´s analysis of compound **2**

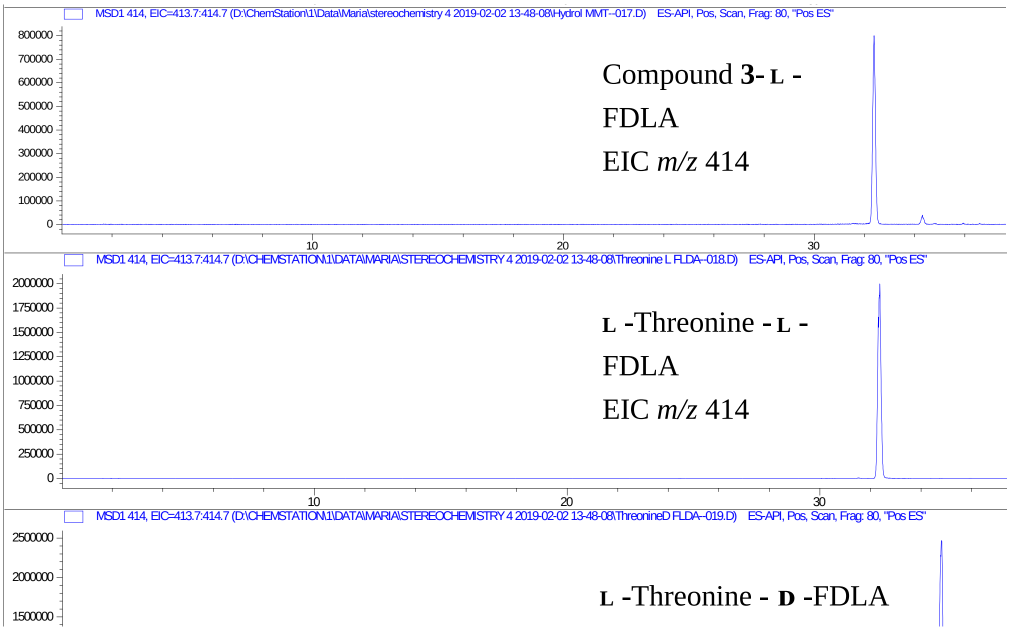

Figure S3 LC-MS of Marfey´s analysis of compound **3**

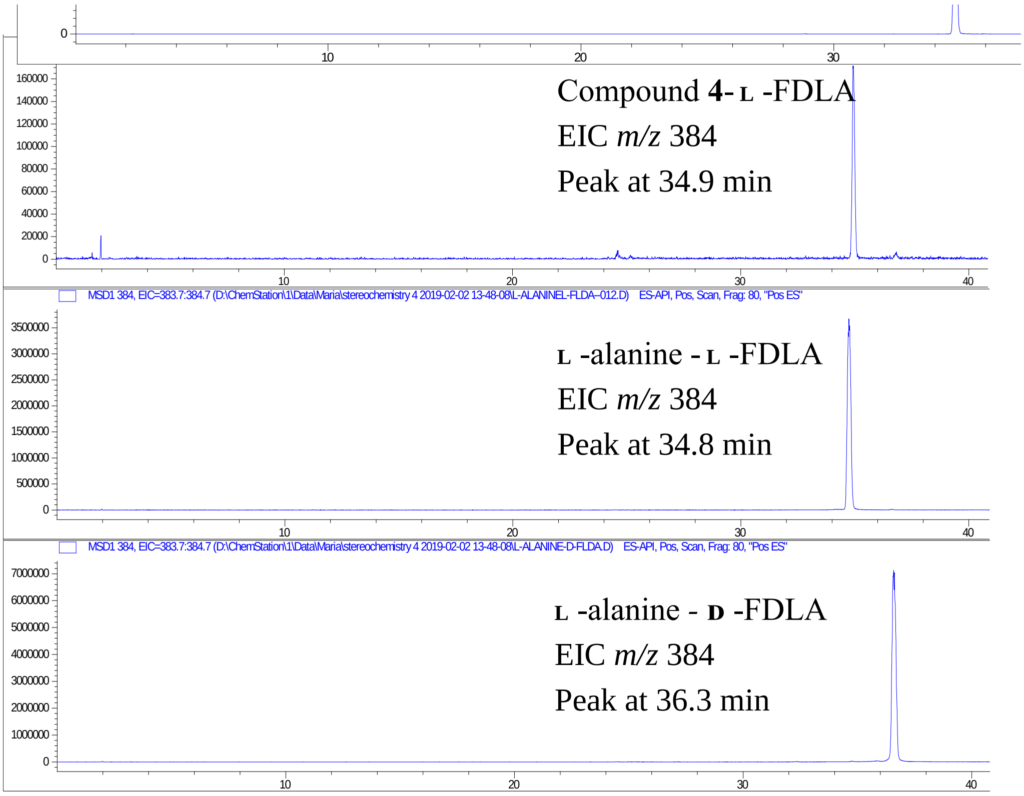

Figure S4 LC-MS of Marfey´s analysis of compound **4**

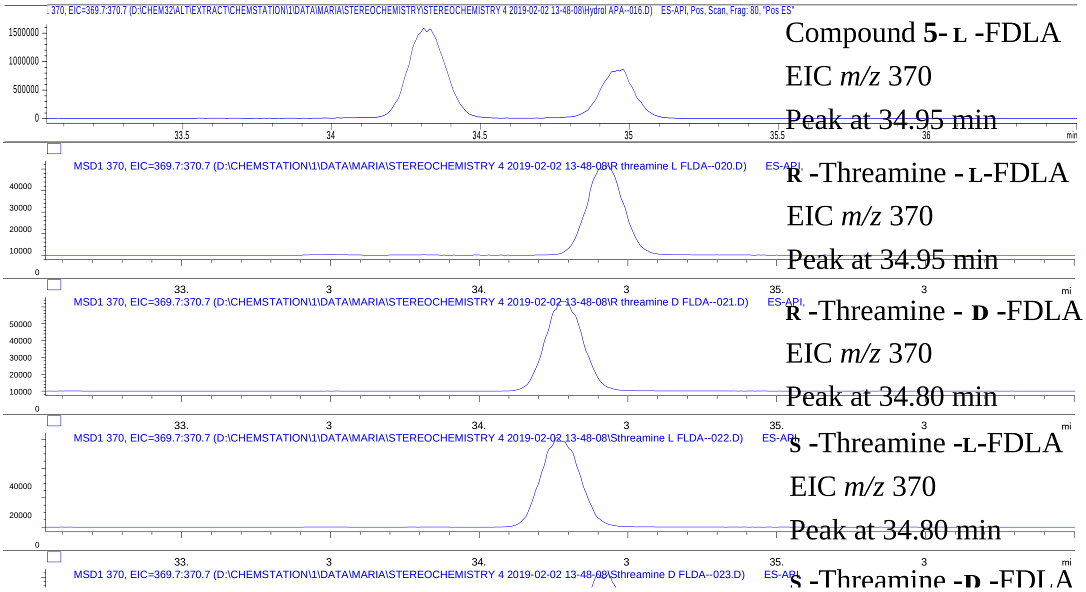

Figure S5 LC-MS of Marfey´s analysis of compound **5**

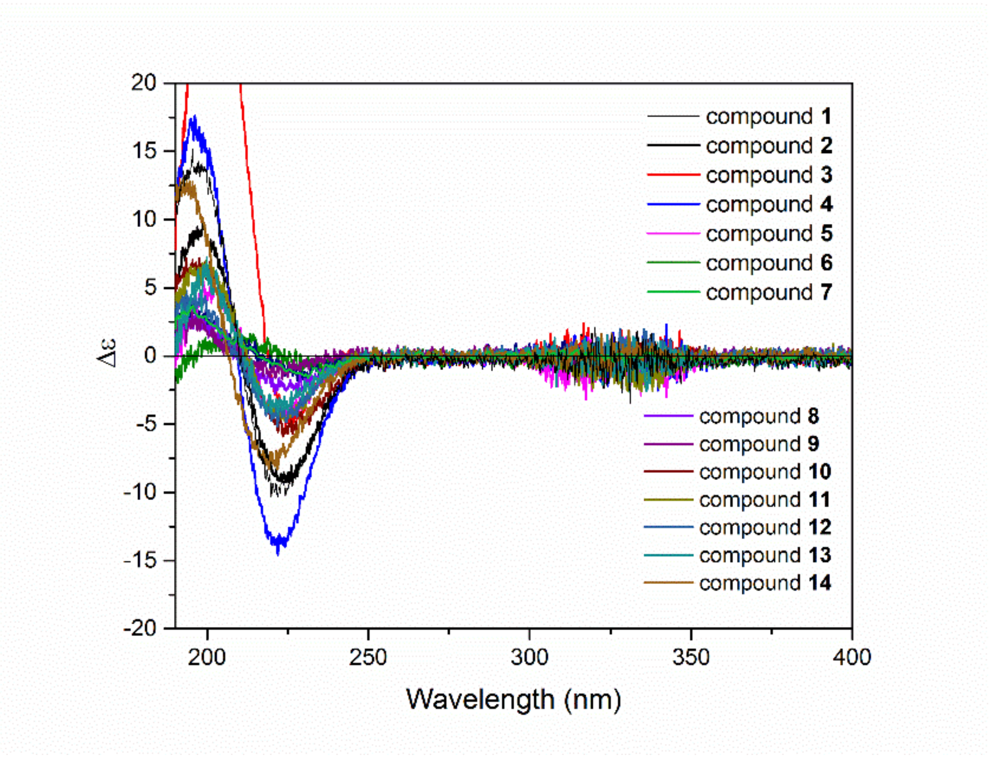

Figure S6. Experimental ECD spectra of isolated compounds.

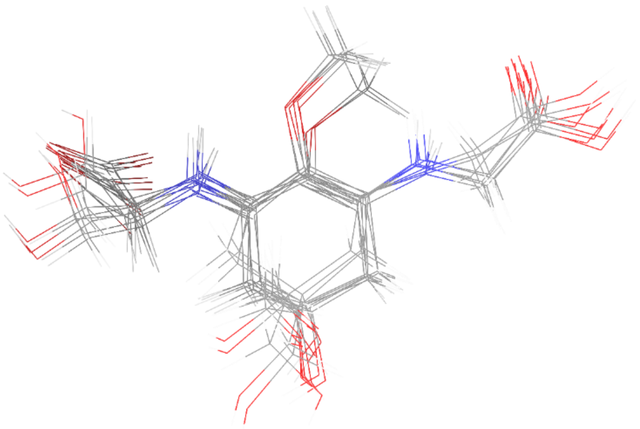

Compound 1 conformers

	Δ	KJ/mol	ΔG/RT	e (-ΔG/RT)	mol		population %
Conformer 1	-1218.210529	0	0	0	1	0.4230	42.29573
Conformer 2	-1218.209515	0.001014	2.662257	1.092846	0.335261	0.1418	14.1801
Conformer 3	-1218.209487	0.001042	2.735771	1.123023	0.325295	0.1376	13.75858
Conformer 4	-1218.20933	0.001199	3.147975	1.292231	0.274657	0.1162	11.61683
Conformer 5	-1218.208695	0.001834	4.815167	1.976607	0.138538	0.0586	5.859584
Conformer 6	-1218.208372	0.002157	5.663203	2.324723	0.097811	0.0414	4.136967
Conformer 7	-1218.208127	0.002402	6.306451	2.588774	0.075112	0.0318	3.17692
Conformer 8	-1218.208083	0.002446	6.421973	2.636195	0.071633	0.0303	3.029783
Conformer 9	-1218.207672	0.002857	7.501053	3.079154	0.045998	0.0195	1.945526
					2.364305	1	100

Figure S7. Overlayered conformers and population of Boltzmann averaged conformers of compound **1.**

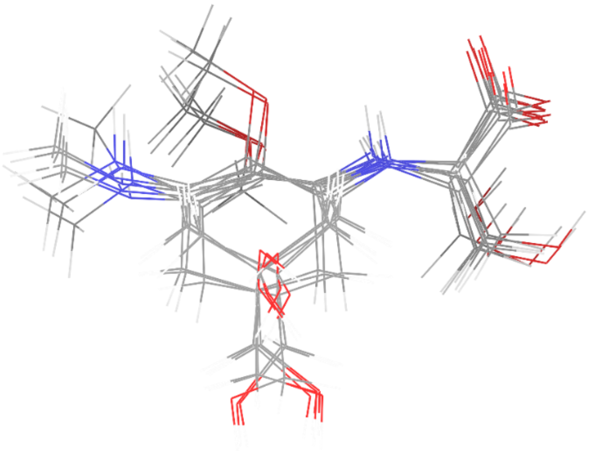

Compound **3** Conformers

	**Δ**	**KJ/mol**	**ΔG/RT**	**e (-ΔG/RT)**	**mol**		**population %**
Conformer 1	-1029.687871	0	0	0	1	0.3779	37.79439
Conformer 2	-1029.687618	0.000253	0.664252	0.272673	0.761342	0.2877	28.77446
Conformer 3	-1029.686828	0.001043	2.738396	1.124101	0.324944	0.1228	12.28108
Conformer 4	-1029.686279	0.001592	4.179796	1.71579	0.179822	0.0680	6.796248
Conformer 5	-1029.686037	0.001834	4.815167	1.976607	0.138538	0.0524	5.235976
Conformer 6	-1029.685999	0.001872	4.914936	2.017562	0.132979	0.0503	5.02587
Conformer 7	-1029.68535	0.002521	6.618886	2.717027	0.066071	0.0250	2.497109
Conformer 8	-1029.684934	0.002937	7.711093	3.165374	0.042198	0.0159	1.594861
					2.645895	1	100

Figure S8. Overlayered conformers and population of Boltzmann averaged conformers of compound **3.**

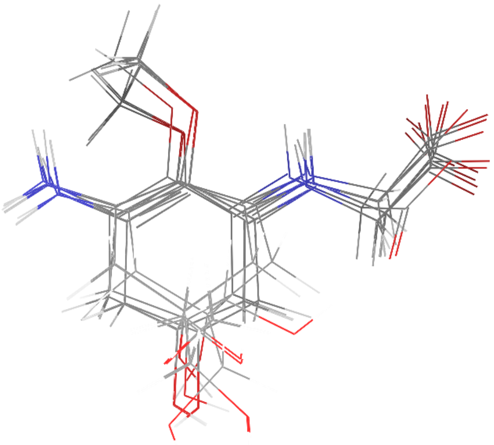

Compound **7** conformers

	Δ	KJ/mol	ΔG/RT	e (-ΔG/RT)	mol		population%
Conformer 1	-875.938525	0	0	0	1	0.5066	50.656
Conformer 2	-875.937141	0.001384	3.633692	1.491617	0.225009	0.1140	11.39804
Conformer 3	-875.937098	0.001427	3.746589	1.53796	0.214819	0.1088	10.88186
Conformer 4	-875.937097	0.001428	3.749214	1.539038	0.214587	0.1087	10.87014
Conformer 5	-875.936251	0.002274	5.970387	2.450821	0.086223	0.0437	4.367701
Conformer 6	-875.93625	0.002275	5.973013	2.451899	0.08613	0.0436	4.362996
Conformer 7	-875.936105	0.00242	6.35371	2.608173	0.073669	0.0373	3.731776
Conformer 8	-875.936105	0.00242	6.35371	2.608173	0.073669	0.0373	3.731776
					1.974106	1.000	100

Figure S9. Overlayered conformers and population of Boltzmann averaged conformers of compounds **7**.

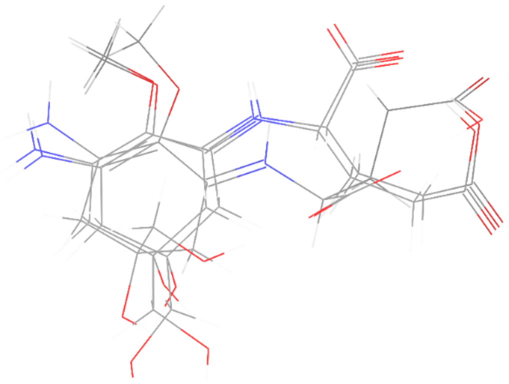

Compound **9** conformers

	Δ	KJ/mol	ΔG/RT	e (-ΔG/RT)	mol		population %
Conformer 1	-1143.028128	0	0	0	1	0.9175	91.75153684
Conformer 2	-1143.025544	0.002584	6.784292	2.784926	0.061733677	0.0566	5.66415974
Conformer 3	-1143.024816	0.003312	8.695656	3.569533	0.028168999	0.0258	2.584548974
					1.08990	1.000	100

Figure S10. Overlayered conformers and population of Boltzmann averaged conformers of compounds **9.**

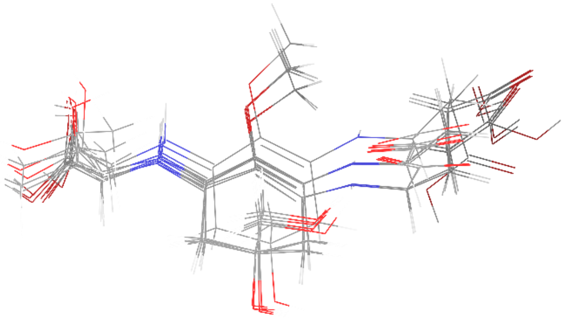

		Δ	KJ/mol	ΔG/RT	e (-ΔG/RT)	mol	
Conformer 1	-1524.428038	0	0	0	1	0.4028	40.27776
Conformer 2	-1524.427026	0.001012	2.657006	1.090691	0.335984	0.1353	13.5327
Conformer 3	-1524.426966	0.001072	2.814536	1.155356	0.314945	0.1269	12.68529
Conformer 4	-1524.426915	0.001123	2.948437	1.210322	0.298101	0.1201	12.00685
Conformer 5	-1524.426667	0.001371	3.599561	1.477606	0.228183	0.0919	9.190714
Conformer 6	-1524.426578	0.00146	3.83323	1.573526	0.207313	0.0835	8.350098
Conformer 7	-1524.425885	0.002153	5.652701	2.320412	0.098233	0.0396	3.956608
					2.48276	1	100

Figure S11. Overlayered conformers and population of Boltzmann averaged conformers of compounds **10.**

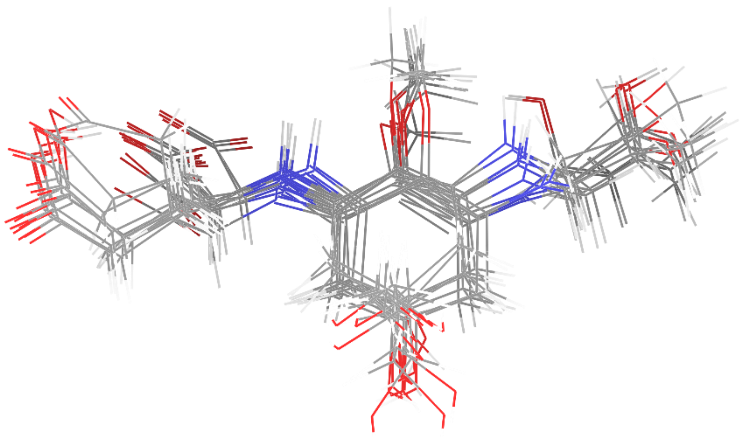

Compound **11** conformers

	Δ	KJ/mol	ΔG/RT	e (-ΔG/RT)	mol		population %
conformer 1	-1319.937126	0	0	0	1.000000	0.2967	29.6735905
conformer 2	-1319.936711	0.000415	1.089582	0.447269	0.639372	0.1897	18.97245198
conformer 3	-1319.936562	0.000564	1.480782	0.607855	0.544517	0.1616	16.15778774
conformer 4	-1319.936491	0.000635	1.667193	0.684376	0.504405	0.1497	14.96750257
conformer 5	-1319.93526	0.001866	4.899183	2.011096	0.133842	0.0397	3.971570986
conformer 6	-1319.935129	0.001997	5.243124	2.152282	0.116219	0.0345	3.448624594
conformer 7	-1319.935121	0.002005	5.264128	2.160904	0.115221	0.0342	3.419018161
conformer 8	-1319.935001	0.002125	5.579187	2.290235	0.101243	0.0300	3.004233644
conformer 9	-1319.934966	0.00216	5.67108	2.327956	0.097495	0.0289	2.893020153
conformer 10	-1319.934514	0.002612	6.857806	2.815103	0.059899	0.0178	1.777405203
conformer 11	-1319.934484	0.002642	6.936571	2.847436	0.057993	0.0172	1.720855965
					3.370204	1.000	100

Figure S12. Overlayered conformers and population of Boltzmann averaged conformers of compounds **11.**

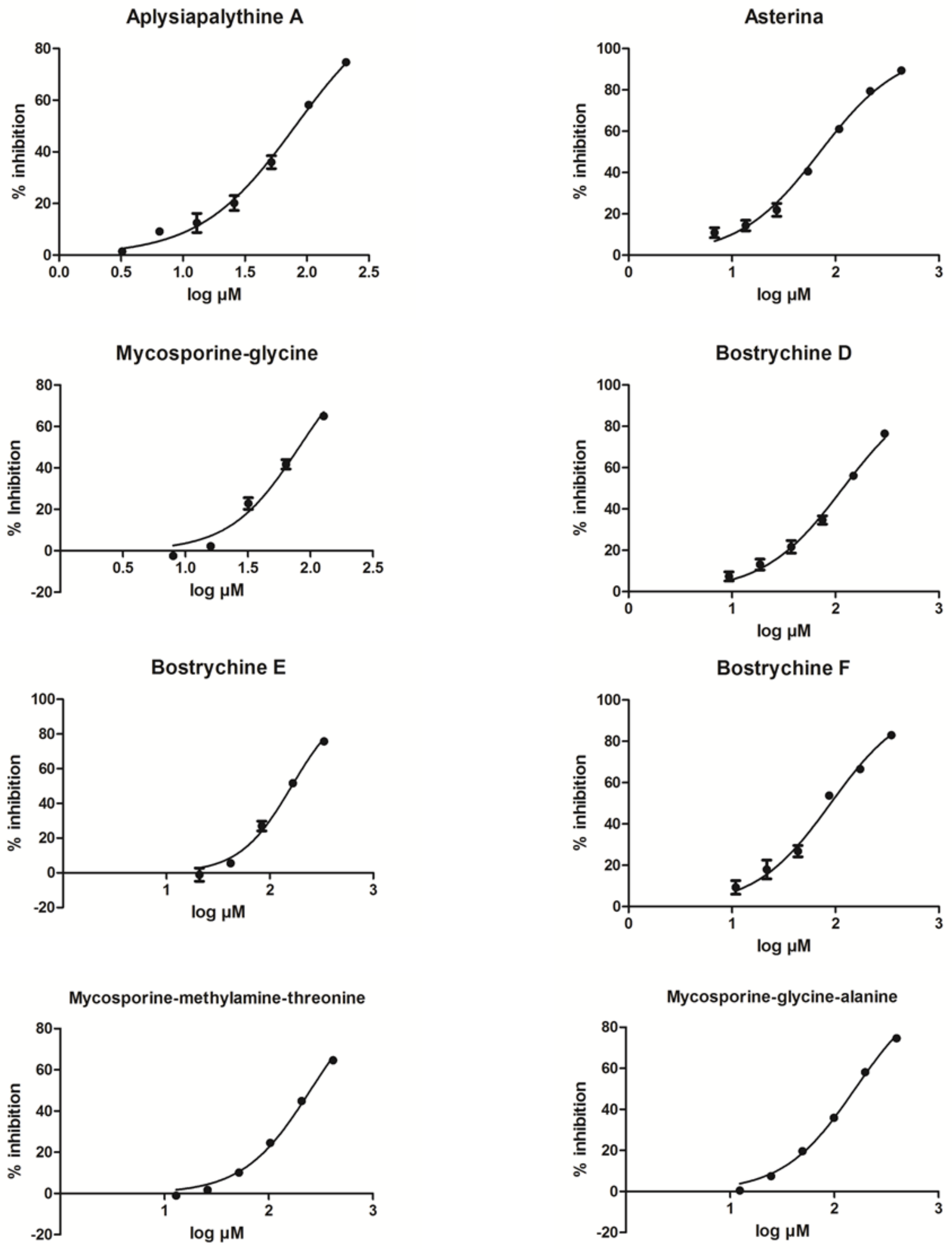

Figure S13. Collagenase inhibitory activity, concentration response curves of all tested MAAs. All data shown as means ± SD (*n* = 3).

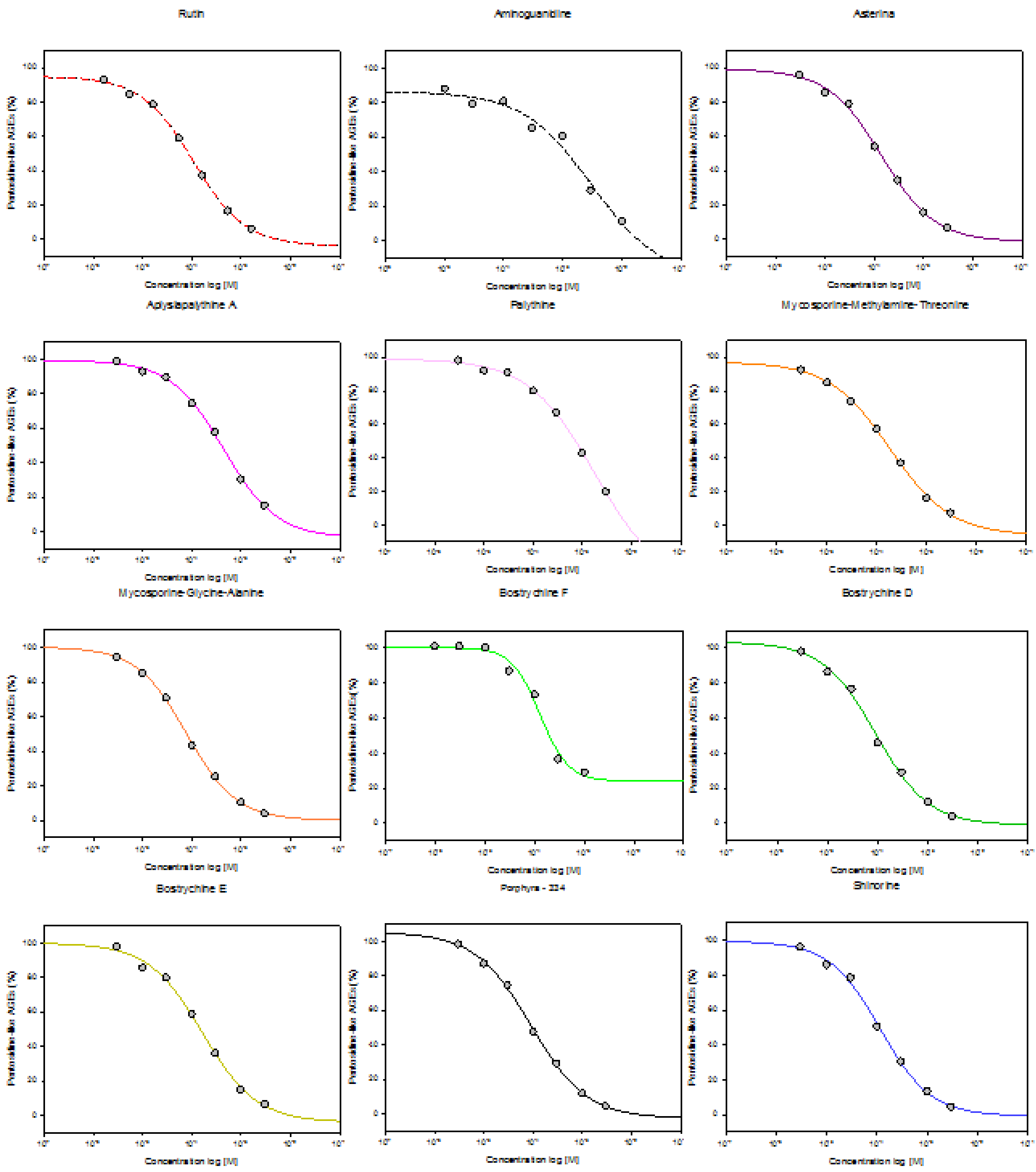

Figure S14. Dose-effect curves for pentosidine-like AGEs formation in the presence of various concentrations of tested MAAs and rutin and aminoguanidine used as reference compounds.

Table S1. Crystallographic data for the hydrate of the shinorine hydrate **1**H.

Moiety Formula	$C_{13}H_{20}N_2O_8 \cdot 1.72 (H_2O)$
Empirical formula	$C_{13}H_{23.44}N_2O_{9.72}$
Formula weight	363.30
Temperature (K)	193(2)
Crystal system	Triclinic
Space group	*P*1
a (Å)	5.4387(3)
b (Å)	11.7023(6)
c (Å)	13.6335(7)
α (°)	105.844(4)
β (°)	99.478(4)
γ (°)	94.044(4)
Unit cell volume ($Å^3$)	817.23(8)
Z / Z′	2 / 2
Reflections collected / *R*int	14277 / 0.0640
Data / restraints / parameters	5597 / 21 / 550
Goodness-of-fit on F^2	1.082
Final *R* indices [$I > 2\sigma(I)$]	*R*1 = 0.0439, *wR*2 = 0.1115
R indices (all data)	*R*1 = 0.0490, *wR*2 = 0.1199
Absolute structure parameter	0.09(14)
Largest diff. peak and hole ($e \cdot Å^{-3}$)	0.329 and -0.226
CCDC no.	1968399

Table S 2. Atomic coordinates (× 10^4) and equivalent isotropic displacement parameters.

($Å^2 \times 10^3$). U_{eq} is defined as one third of the trace of the orthogonalized U^{ij} tensor.

	**x**	**y**	**z**	U_{eq}
C(1A)	5844(7)	1280(4)	9230(3)	23(1)
C(2A)	5520(8)	1993(3)	8442(3)	24(1)
C(3A)	4329(7)	1251(4)	7365(3)	20(1)
C(4A)	4014(7)	11(3)	7112(3)	20(1)
C(5A)	5159(7)	-614(4)	7765(3)	22(1)
C(6A)	6809(8)	99(4)	8781(3)	25(1)
O(7A)	3529(6)	951(3)	9500(3)	30(1)
C(8A)	7613(8)	2036(4)	10224(3)	29(1)
O(9A)	9990(6)	2395(3)	10039(3)	36(1)
N(10A)	3591(6)	1805(3)	6662(3)	22(1)
C(11A)	3832(7)	3092(3)	6855(3)	22(1)
C(12A)	4078(8)	3411(4)	5856(3)	28(1)
O(13A)	1810(7)	3035(3)	5115(2)	34(1)
O(14A)	2592(5)	-627(2)	6152(2)	23(1)
C(15A)	6(8)	-915(4)	6216(4)	32(1)
N(16A)	4921(7)	-1795(3)	7462(3)	24(1)
C(17A)	5820(9)	-2572(4)	8091(3)	28(1)
C(18A)	7357(8)	-3499(4)	7552(4)	28(1)
O(19A)	7579(8)	-3582(3)	6659(3)	45(1)
C(20A)	1595(8)	3617(4)	7282(3)	25(1)
O(21A)	1807(6)	4772(3)	7461(3)	37(1)
O(22A)	8245(7)	-4146(3)	8104(3)	39(1)
O(23A)	-109(7)	3018(4)	7420(4)	53(1)

C(1B)	1868(7)	8775(4)	1246(3)	23(1)
C(2B)	2974(8)	8167(4)	2038(3)	25(1)
C(3B)	2528(7)	8761(4)	3113(3)	23(1)
C(4B)	2006(8)	9929(4)	3367(3)	24(1)
C(5B)	2171(7)	10652(4)	2710(3)	23(1)
C(6B)	2660(8)	10113(4)	1638(3)	29(1)
O(7B)	-821(5)	8609(3)	1084(2)	29(1)
C(8B)	2726(8)	8240(4)	223(3)	30(1)
O(9B)	1558(7)	8657(4)	-602(3)	32(1)
O(9')	2300(30)	6979(9)	-142(10)	28(3)
N(10B)	2749(7)	8165(3)	3822(3)	25(1)
C(11B)	2623(7)	6867(3)	3580(3)	23(1)
C(12B)	3753(8)	6509(4)	4532(4)	28(1)
O(13B)	2344(6)	6836(3)	5336(2)	32(1)
O(14B)	1437(5)	10436(2)	4342(2)	24(1)
C(15B)	-1246(9)	10444(5)	4277(4)	35(1)
N(16B)	1917(7)	11796(3)	3045(3)	25(1)
C(17B)	2252(8)	12692(4)	2502(3)	27(1)
C(18B)	4874(8)	13371(4)	2821(3)	27(1)
O(19B)	5247(6)	14142(3)	2337(3)	36(1)
C(20B)	-111(8)	6314(4)	3148(3)	25(1)
O(21B)	-365(6)	5163(3)	2856(3)	39(1)
O(22B)	6405(7)	13182(4)	3508(3)	51(1)
O(23B)	-1804(7)	6909(3)	3080(4)	58(1)
O(1W)	-779(6)	4875(3)	5168(3)	31(1)
O(1C)	-2140(20)	6059(7)	176(6)	89(3)
O(1D)	-3500(30)	6186(17)	755(18)	82(6)
O(2C)	11800(30)	4697(7)	10658(7)	89(4)
O(2D)	4310(30)	14822(18)	547(13)	108(6)
O(3D)	-830(30)	4631(11)	9687(9)	72(3)

Table S3. Bond lengths [Å] and angles [°].

**C(1A)-O(7A)**	**1.425(5)**
C(1A)-C(6A)	1.519(6)
C(1A)-C(2A)	1.526(6)
C(1A)-C(8A)	1.530(6)
C(2A)-C(3A)	1.497(6)
C(2A)-H(2A1)	0.9900
C(2A)-H(2A2)	0.9900
C(3A)-N(10A)	1.323(5)
C(3A)-C(4A)	1.387(6)
C(4A)-O(14A)	1.386(5)
C(4A)-C(5A)	1.397(6)
C(5A)-N(16A)	1.320(5)
C(5A)-C(6A)	1.508(6)
C(6A)-H(6A1)	0.9900
C(6A)-H(6A2)	0.9900

O(7A)-H(7A)	0.840(15)
C(8A)-O(9A)	1.414(5)
C(8A)-H(8A1)	0.9900
C(8A)-H(8A2)	0.9900
O(9A)-H(9A)	0.847(15)
N(10A)-C(11A)	1.450(5)
N(10A)-H(10A)	0.867(15)
C(11A)-C(20A)	1.530(6)
C(11A)-C(12A)	1.531(6)
C(11A)-H(11A)	10.000
C(12A)-O(13A)	1.416(6)
C(12A)-H(12A)	0.9900
C(12A)-H(12B)	0.9900
O(13A)-H(13A)	0.8400
O(14A)-C(15A)	1.445(5)
C(15A)-H(15A)	0.9800
C(15A)-H(15B)	0.9800
C(15A)-H(15C)	0.9800
N(16A)-C(17A)	1.467(6)
N(16A)-H(16A)	0.882(15)
C(17A)-C(18A)	1.521(6)
C(17A)-H(17A)	0.9900
C(17A)-H(17B)	0.9900
C(18A)-O(19A)	1.221(6)
C(18A)-O(22A)	1.271(6)
C(20A)-O(23A)	1.193(6)
C(20A)-O(21A)	1.300(6)
O(21A)-H(21A)	0.838(15)
C(1B)-O(7B)	1.434(5)
C(1B)-C(6B)	1.514(6)
C(1B)-C(2B)	1.517(6)
C(1B)-C(8B)	1.527(6)
C(2B)-C(3B)	1.508(5)
C(2B)-H(2B1)	0.9900
C(2B)-H(2B2)	0.9900
C(3B)-N(10B)	1.333(6)
C(3B)-C(4B)	1.377(6)
C(4B)-O(14B)	1.394(5)
C(4B)-C(5B)	1.400(6)
C(5B)-N(16B)	1.316(5)
C(5B)-C(6B)	1.501(6)
C(6B)-H(6B1)	0.9900

C(6B)-H(6B2)	0.9900
O(7B)-H(7B)	0.843(15)
C(8B)-O(9')	1.412(10)
C(8B)-O(9B)	1.420(6)
C(8B)-H(8B1)	0.9900
C(8B)-H(8B2)	0.9900
C(8B)-H(8B3)	0.9900
C(8B)-H(8B4)	0.9900
O(9B)-H(9B)	0.8400
O(9')-H(9')	0.8400
N(10B)-C(11B)	1.458(5)
N(10B)-H(10B)	0.875(15)
C(11B)-C(12B)	1.520(6)
C(11B)-C(20B)	1.531(6)
C(11B)-H(11B)	10.000
C(12B)-O(13B)	1.422(5)
C(12B)-H(12C)	0.9900
C(12B)-H(12D)	0.9900
O(13B)-H(13B)	0.8400
O(14B)-C(15B)	1.448(5)
C(15B)-H(15D)	0.9800
C(15B)-H(15E)	0.9800
C(15B)-H(15F)	0.9800
N(16B)-C(17B)	1.456(5)
N(16B)-H(16B)	0.882(15)
C(17B)-C(18B)	1.518(6)
C(17B)-H(17C)	0.9900
C(17B)-H(17D)	0.9900
C(18B)-O(22B)	1.226(6)
C(18B)-O(19B)	1.278(5)
C(20B)-O(23B)	1.200(6)
C(20B)-O(21B)	1.286(6)
O(21B)-H(21B)	0.839(15)
O(1W)-H(1W1)	0.842(15)
O(1W)-H(1W2)	0.846(15)
O(7A)-C(1A)-C(6A)	104.7(3)
O(7A)-C(1A)-C(2A)	112.5(3)
C(6A)-C(1A)-C(2A)	110.9(3)
O(7A)-C(1A)-C(8A)	108.0(3)
C(6A)-C(1A)-C(8A)	111.4(3)
C(2A)-C(1A)-C(8A)	109.3(3)
C(3A)-C(2A)-C(1A)	113.6(3)

C(3A)-C(2A)-H(2A1)	108.8
C(1A)-C(2A)-H(2A1)	108.8
C(3A)-C(2A)-H(2A2)	108.8
C(1A)-C(2A)-H(2A2)	108.8
H(2A1)-C(2A)-H(2A2)	107.7
N(10A)-C(3A)-C(4A)	120.9(4)
N(10A)-C(3A)-C(2A)	118.4(4)
C(4A)-C(3A)-C(2A)	120.7(3)
O(14A)-C(4A)-C(3A)	118.3(3)
O(14A)-C(4A)-C(5A)	119.0(3)
C(3A)-C(4A)-C(5A)	122.7(4)
N(16A)-C(5A)-C(4A)	120.3(4)
N(16A)-C(5A)-C(6A)	121.5(4)
C(4A)-C(5A)-C(6A)	118.1(3)
C(5A)-C(6A)-C(1A)	111.5(3)
C(5A)-C(6A)-H(6A1)	109.3
C(1A)-C(6A)-H(6A1)	109.3
C(5A)-C(6A)-H(6A2)	109.3
C(1A)-C(6A)-H(6A2)	109.3
H(6A1)-C(6A)-H(6A2)	108.0
C(1A)-O(7A)-H(7A)	112(7)
O(9A)-C(8A)-C(1A)	112.3(3)
O(9A)-C(8A)-H(8A1)	109.1
C(1A)-C(8A)-H(8A1)	109.1
O(9A)-C(8A)-H(8A2)	109.1
C(1A)-C(8A)-H(8A2)	109.1
H(8A1)-C(8A)-H(8A2)	107.9
C(8A)-O(9A)-H(9A)	108(5)
C(3A)-N(10A)-C(11A)	124.8(3)
C(3A)-N(10A)-H(10A)	120(3)
C(11A)-N(10A)-H(10A)	116(3)
N(10A)-C(11A)-C(20A)	111.1(3)
N(10A)-C(11A)-C(12A)	110.1(3)
C(20A)-C(11A)-C(12A)	110.4(3)
N(10A)-C(11A)-H(11A)	108.4
C(20A)-C(11A)-H(11A)	108.4
C(12A)-C(11A)-H(11A)	108.4
O(13A)-C(12A)-C(11A)	110.7(3)
O(13A)-C(12A)-H(12A)	109.5
C(11A)-C(12A)-H(12A)	109.5
O(13A)-C(12A)-H(12B)	109.5
C(11A)-C(12A)-H(12B)	109.5

H(12A)-C(12A)-H(12B)	108.1
C(12A)-O(13A)-H(13A)	109.5
C(4A)-O(14A)-C(15A)	111.6(3)
O(14A)-C(15A)-H(15A)	109.5
O(14A)-C(15A)-H(15B)	109.5
H(15A)-C(15A)-H(15B)	109.5
O(14A)-C(15A)-H(15C)	109.5
H(15A)-C(15A)-H(15C)	109.5
H(15B)-C(15A)-H(15C)	109.5
C(5A)-N(16A)-C(17A)	126.5(4)
C(5A)-N(16A)-H(16A)	120(4)
C(17A)-N(16A)-H(16A)	114(4)
N(16A)-C(17A)-C(18A)	112.9(3)
N(16A)-C(17A)-H(17A)	109.0
C(18A)-C(17A)-H(17A)	109.0
N(16A)-C(17A)-H(17B)	109.0
C(18A)-C(17A)-H(17B)	109.0
H(17A)-C(17A)-H(17B)	107.8
O(19A)-C(18A)-O(22A)	126.6(4)
O(19A)-C(18A)-C(17A)	119.6(4)
O(22A)-C(18A)-C(17A)	113.8(4)
O(23A)-C(20A)-O(21A)	125.6(4)
O(23A)-C(20A)-C(11A)	123.0(4)
O(21A)-C(20A)-C(11A)	111.4(3)
C(20A)-O(21A)-H(21A)	103(6)
O(7B)-C(1B)-C(6B)	106.5(3)
O(7B)-C(1B)-C(2B)	110.2(3)
C(6B)-C(1B)-C(2B)	110.7(4)
O(7B)-C(1B)-C(8B)	109.7(3)
C(6B)-C(1B)-C(8B)	110.5(3)
C(2B)-C(1B)-C(8B)	109.2(3)
C(3B)-C(2B)-C(1B)	112.6(3)
C(3B)-C(2B)-H(2B1)	109.1
C(1B)-C(2B)-H(2B1)	109.1
C(3B)-C(2B)-H(2B2)	109.1
C(1B)-C(2B)-H(2B2)	109.1
H(2B1)-C(2B)-H(2B2)	107.8
N(10B)-C(3B)-C(4B)	120.6(4)
N(10B)-C(3B)-C(2B)	119.5(4)
C(4B)-C(3B)-C(2B)	119.8(4)
C(3B)-C(4B)-O(14B)	118.9(4)
C(3B)-C(4B)-C(5B)	122.5(4)

O(14B)-C(4B)-C(5B)	118.4(3)
N(16B)-C(5B)-C(4B)	119.5(4)
N(16B)-C(5B)-C(6B)	121.1(4)
C(4B)-C(5B)-C(6B)	119.4(4)
C(5B)-C(6B)-C(1B)	112.4(3)
C(5B)-C(6B)-H(6B1)	109.1
C(1B)-C(6B)-H(6B1)	109.1
C(5B)-C(6B)-H(6B2)	109.1
C(1B)-C(6B)-H(6B2)	109.1
H(6B1)-C(6B)-H(6B2)	107.8
C(1B)-O(7B)-H(7B)	98(7)
O(9')-C(8B)-C(1B)	114.3(6)
O(9B)-C(8B)-C(1B)	113.3(3)
O(9B)-C(8B)-H(8B1)	108.9
C(1B)-C(8B)-H(8B1)	108.9
O(9B)-C(8B)-H(8B2)	108.9
C(1B)-C(8B)-H(8B2)	108.9
H(8B1)-C(8B)-H(8B2)	107.7
O(9')-C(8B)-H(8B3)	108.7
C(1B)-C(8B)-H(8B3)	108.7
O(9')-C(8B)-H(8B4)	108.7
C(1B)-C(8B)-H(8B4)	108.7
H(8B3)-C(8B)-H(8B4)	107.6
C(8B)-O(9B)-H(9B)	109.5
C(8B)-O(9')-H(9')	109.5
C(3B)-N(10B)-C(11B)	124.4(3)
C(3B)-N(10B)-H(10B)	113(3)
C(11B)-N(10B)-H(10B)	118(3)
N(10B)-C(11B)-C(12B)	110.6(3)
N(10B)-C(11B)-C(20B)	109.0(3)
C(12B)-C(11B)-C(20B)	112.3(3)
N(10B)-C(11B)-H(11B)	108.3
C(12B)-C(11B)-H(11B)	108.3
C(20B)-C(11B)-H(11B)	108.3
O(13B)-C(12B)-C(11B)	111.5(4)
O(13B)-C(12B)-H(12C)	109.3
C(11B)-C(12B)-H(12C)	109.3
O(13B)-C(12B)-H(12D)	109.3
C(11B)-C(12B)-H(12D)	109.3
H(12C)-C(12B)-H(12D)	108.0
C(12B)-O(13B)-H(13B)	109.5
C(4B)-O(14B)-C(15B)	111.1(3)

O(14B)-C(15B)-H(15D)	109.5
O(14B)-C(15B)-H(15E)	109.5
H(15D)-C(15B)-H(15E)	109.5
O(14B)-C(15B)-H(15F)	109.5
H(15D)-C(15B)-H(15F)	109.5
H(15E)-C(15B)-H(15F)	109.5
C(5B)-N(16B)-C(17B)	126.6(4)
C(5B)-N(16B)-H(16B)	117(3)
C(17B)-N(16B)-H(16B)	115(3)
N(16B)-C(17B)-C(18B)	112.7(3)
N(16B)-C(17B)-H(17C)	109.1
C(18B)-C(17B)-H(17C)	109.1
N(16B)-C(17B)-H(17D)	109.1
C(18B)-C(17B)-H(17D)	109.1
H(17C)-C(17B)-H(17D)	107.8
O(22B)-C(18B)-O(19B)	125.5(4)
O(22B)-C(18B)-C(17B)	120.3(4)
O(19B)-C(18B)-C(17B)	114.2(4)
O(23B)-C(20B)-O(21B)	124.6(4)
O(23B)-C(20B)-C(11B)	122.5(4)
O(21B)-C(20B)-C(11B)	112.9(4)
C(20B)-O(21B)-H(21B)	118(5)
H(1W1)-O(1W)-H(1W2)	105(4)

Symmetry transformations used to generate equivalent atoms:

Table S4. Anisotropic displacement parameters (Å^2 × 10^3). The anisotropic displacement factor exponent takes the form: $-2\pi^2$ [h^2 a^{*2} U^{11} + ... + 2 h k a^* b^* U^{12}].

	U^{11}	U^{22}	U^{33}	U^{23}	U^{13}	U^{12}
C(1A)	21(2)	31(2)	19(2)	9(2)	3(2)	5(2)
C(2A)	26(2)	21(2)	23(2)	5(2)	6(2)	2(1)
C(3A)	16(2)	29(2)	17(2)	9(2)	3(2)	5(1)
C(4A)	21(2)	24(2)	16(2)	6(2)	4(2)	2(1)
C(5A)	20(2)	27(2)	21(2)	10(2)	10(2)	4(2)
C(6A)	23(2)	30(2)	24(2)	12(2)	3(2)	6(2)
O(7A)	28(2)	38(2)	32(2)	17(1)	11(1)	7(1)
C(8A)	32(2)	32(2)	22(2)	7(2)	3(2)	8(2)
O(9A)	29(2)	34(2)	39(2)	5(1)	2(1)	0(1)
N(10A)	25(2)	20(2)	18(2)	5(1)	0(1)	1(1)
C(11A)	23(2)	21(2)	23(2)	8(2)	2(2)	2(1)

C(12A)	34(2)	29(2)	26(2)	13(2)	11(2)	6(2)
O(13A)	51(2)	32(2)	22(2)	10(1)	3(1)	15(1)
O(14A)	26(1)	24(1)	20(1)	8(1)	5(1)	-2(1)
C(15A)	26(2)	38(2)	30(2)	10(2)	2(2)	-1(2)
N(16A)	32(2)	19(2)	24(2)	9(1)	7(2)	4(1)
C(17A)	39(2)	23(2)	29(2)	13(2)	13(2)	9(2)
C(18A)	31(2)	24(2)	32(2)	10(2)	10(2)	5(2)
O(19A)	67(2)	47(2)	32(2)	17(2)	23(2)	29(2)
C(20A)	28(2)	31(2)	17(2)	11(2)	5(2)	3(2)
O(21A)	36(2)	26(2)	52(2)	8(1)	15(2)	8(1)
O(22A)	47(2)	37(2)	41(2)	20(2)	15(2)	21(1)
O(23A)	42(2)	45(2)	95(3)	38(2)	42(2)	16(2)
C(1B)	21(2)	28(2)	20(2)	10(2)	3(2)	3(2)
C(2B)	27(2)	30(2)	22(2)	12(2)	7(2)	6(2)
C(3B)	20(2)	25(2)	23(2)	10(2)	3(2)	-4(1)
C(4B)	26(2)	25(2)	20(2)	7(2)	6(2)	-2(2)
C(5B)	24(2)	22(2)	21(2)	7(2)	2(2)	-4(1)
C(6B)	35(2)	32(2)	25(2)	15(2)	9(2)	4(2)
O(7B)	25(1)	32(2)	29(2)	8(1)	5(1)	4(1)
C(8B)	32(2)	36(2)	23(2)	12(2)	7(2)	10(2)
O(9B)	39(2)	35(2)	23(2)	11(2)	5(2)	8(2)
N(10B)	31(2)	25(2)	22(2)	11(1)	9(2)	3(1)
C(11B)	24(2)	22(2)	24(2)	10(2)	8(2)	0(2)
C(12B)	31(2)	28(2)	29(2)	14(2)	5(2)	0(2)
O(13B)	45(2)	25(2)	26(2)	10(1)	10(1)	-5(1)
O(14B)	29(1)	27(1)	19(1)	8(1)	7(1)	2(1)
C(15B)	30(2)	45(3)	32(2)	12(2)	10(2)	2(2)
N(16B)	34(2)	24(2)	19(2)	11(1)	2(2)	1(1)
C(17B)	33(2)	21(2)	28(2)	13(2)	1(2)	0(2)
C(18B)	32(2)	27(2)	20(2)	9(2)	-1(2)	-1(2)
O(19B)	37(2)	34(2)	39(2)	18(1)	2(2)	-6(1)
C(20B)	28(2)	28(2)	24(2)	14(2)	7(2)	2(2)
O(21B)	33(2)	25(2)	54(2)	7(1)	0(2)	1(1)
O(22B)	46(2)	56(2)	53(2)	33(2)	-11(2)	-13(2)
O(23B)	26(2)	33(2)	114(4)	25(2)	5(2)	4(1)
O(1W)	33(2)	30(2)	29(2)	7(1)	7(1)	5(1)
O(1C)	160(9)	55(4)	53(4)	8(3)	33(5)	22(5)
O(1D)	69(10)	74(11)	86(14)	9(9)	-21(10)	34(9)
O(2C)	172(11)	34(4)	39(4)	12(3)	-34(6)	-1(5)
O(2D)	67(8)	170(16)	106(12)	97(12)	-7(8)	-34(9)
O(3D)	89(8)	70(7)	58(6)	24(5)	13(6)	-4(6)

Table S5. Hydrogen coordinates (× 10^4) and isotropic displacement parameters (Å^2 × 10^3).

	x	y	z	U^{eq}
H(2A1)	4474	2636	8675	14(10)
H(2A2)	7187	2378	8426	20(11)
H(6A1)	8539	258	8669	23(11)
H(6A2)	6870	-374	9283	25(12)
H(7A)	2590(140)	1500(60)	9550(80)	90(30)
H(8A1)	6840	2756	10535	51(17)
H(8A2)	7838	1568	10730	49(16)
H(9A)	10150(150)	3150(16)	10160(60)	70(20)
H(10A)	2980(70)	1390(30)	6026(16)	10(9)
H(11A)	5394	3449	7384	25(12)
H(12A)	4500	4288	6016	33(13)
H(12B)	5458	3020	5563	26(12)
H(13A)	1025	3626	5106	32(14)
H(15A)	-63	-1381	6711	44(16)
H(15B)	-932	-1385	5530	44(16)
H(15C)	-741	-174	6451	33(13)
H(16A)	4050(80)	-2190(40)	6840(20)	30(13)
H(17A)	4359	-2988	8254	46(16)
H(17B)	6862	-2071	8756	60(20)
H(21A)	560(100)	4960(70)	7730(60)	70(20)
H(2B1)	2220	7320	1817	18(10)
H(2B2)	4804	8179	2060	42(15)
H(6B1)	4475	10274	1644	43(15)
H(6B2)	1735	10501	1155	24(12)
H(7B)	-1020(180)	7856(14)	870(70)	90(30)
H(8B1)	4568	8438	329	35
H(8B2)	2352	7358	24	35
H(8B3)	1838	8571	-312	35
H(8B4)	4545	8495	316	35
H(9B)	1894	9404	-444	47
H(9')	752	6754	-239	42
H(10B)	2340(90)	8560(40)	4410(20)	19(11)
H(11B)	3627	6580	3028	33(13)
H(12C)	3800	5633	4336	24(12)
H(12D)	5501	6903	4793	27(12)
H(13B)	1362	6241	5319	29(13)
H(15D)	-1896	10854	3769	56(18)
H(15E)	-1586	10864	4959	41(15)
H(15F)	-2072	9619	4060	41(15)
H(16B)	1800(100)	12080(40)	3702(16)	25(12)
H(17C)	1014	13267	2646	33(13)
H(17D)	1912	12293	1745	28(12)
H(21B)	-1820(60)	4790(60)	2670(60)	70(20)
H(1W1)	-1600(110)	5220(60)	5610(40)	70(20)
H(1W2)	-1840(90)	4600(60)	4610(30)	56(19)

Table S6. Hydrogen bonds [Å and °].

D—H···*A*	d_{D-H}	$d_{H\cdots A}$	$d_{D\cdots A}$	<(*DHA*)
O(7A)-H(7A)...O(9A)#1	0.840(15)	1.92(4)	2.710(4)	156(9)
O(9A)-H(9A)...O(2C)	0.847(15)	1.86(4)	2.655(9)	156(8)
O(9A)-H(9A)...O(3D)#2	0.847(15)	2.08(5)	2.838(14)	148(8)
N(10A)-H(10A)...O(14B)#3	0.867(15)	2.248(16)	3.113(4)	176(4)
O(13A)-H(13A)...O(1W)	0.84	1.81	2.646(4)	175.5
N(16A)-H(16A)...O(13B)#3	0.882(15)	2.078(17)	2.957(5)	174(5)
O(21A)-H(21A)...O(22A)#4	0.838(15)	1.74(4)	2.534(4)	157(8)
O(7B)-H(7B)...O(1C)	0.843(15)	2.06(3)	2.889(8)	169(10)
O(7B)-H(7B)...O(1D)	0.843(15)	2.25(6)	2.98(2)	145(9)
N(10B)-H(10B)...O(14A)#5	0.875(15)	2.29(2)	3.123(4)	159(4)
N(10B)-H(10B)...O(14B)	0.875(15)	2.30(5)	2.734(5)	110(4)
O(13B)-H(13B)...O(1W)	0.84	1.85	2.691(4)	175.3
N(16B)-H(16B)...O(13A)#5	0.882(15)	1.943(19)	2.812(5)	168(5)
O(21B)-H(21B)...O(19B)#6	0.839(15)	1.652(16)	2.491(4)	177(8)
O(1W)-H(1W1)...O(19A)#4	0.842(15)	1.85(3)	2.648(4)	159(7)
O(1W)-H(1W2)...O(22B)#6	0.846(15)	1.97(4)	2.716(5)	147(6)
